# Periscope

**Published:** 1881-11

**Authors:** 


### PERISCOPE.

New York Medical Times, May:—Dr. Conlyn reports a case of chronic cystitis after venereal disease, in which the main symptoms were: *constant urging to urinate; passes but a small quantity each time with great burning; severe pains from bladder to kidneys;* sediment of muco-purulent matter. *Cantharis* (3) relieved considerably. *Urging* became more intense. Constant bearing down sensation. Burning remains a long time after urinating with *feeling as if more would pass.* *Nux vomica* (3) cured.

Medical Investigator, Aug.:—Dr. McClanahan reports cures of diabetes and eneuresis with *Rhus aromatica.* His indications are not very clear. They are as follows: profuse stools, cool sallow skin, small feeble pulse, emaciation, flabby abdomen, tongue pale, trembling and moist, trembling of lower limbs, sense of lassitude.

Dr. Morgan rearranges a translation by "S. L." of remedies for rheumatism. Giving the symptoms of *Sepia* with "modalities," and comparisons with other remedies. It is well done. He has done the same with *Nat. mur.* in same disease. The whole thing could be copied into the physician's note-book with advantage. Dr. Morgan also gives some "seasonable indications" for a few remedies in summer bowel complaints. These also are useful.

Medical Advance, Aug.:—Dr. Camp reports a very interesting case of intermittent fever, which he first treated after a mongrel fashion for a period of thirty days. He then, very sensibly, tried to clear up the case by administration of *Sulphur*. The result was that a violent chill was brought on with symptoms, as follows: Chill lasting two hours; chill commencing in lumbar region, and spreading up and down spine to extremities; chill with thirst; *chill much worse from uncovering, even a hand. Desire to be heavily covered in bed. Hepar* was given, which *prevented* the next chill, and in ten days she was perfectly well.

Dr. McNeil translates the following: A man threatened with phthisis. Violent cough with aphonia. *Cough is worse from midnight until 5* A. M. *Oppression of chest, which compels him to sit, bent forward. Cough compels him to sit bent forward. Kali carb.* relieved at once. At the same time this remedy caused the disappearance of two lipomata under lower angle of each shoulder blade.

A woman had had an attack of peritonitis, which had been treated by an old school doctor. She now had exudation in right side of pelvic cavity, weakness, small pulse, sleeplessness, constipation, etc, *Kali carb.* caused decrease of swelling and general improvement.

A man suffered for two years with asthma and emphysema. The paroxysms occurred every eight days, *beginning at two or three o'clock* A. M., *and lasting about two hours. Kali carb.* caused immediate improvement. A woman had violent toothache, *worse in the early morning hours. Kali carb.* cured.

The Clinique, July 15:—The "Bureau of Diseases of Children" reports four cases of diarrhœa as follow: First, a female infant, stools of water, mucus, and undigested matter. *Heavy, white coating on tongue. Antimon. crud.* (3) cured. No. 2. Diarrhœa *with long-continued vomiting, tongue heavily coated white.* Stools, greenish water and mucus. *Antimon. crud.* (3) cured. No. 3. Diarrhœa of watery stools mixed with *curds of casein* and shreds of mucus. *Child could not bear to be touched or looked at. Tongue covered with heavy white coating. Aggravation of stool from cold water. Antimon. crud.* (3) cured. No. 4. Child of five years had *diarrhœa after drinking cold water. Vomiting of ingesta. Tongue had heavy white coating. Antimon. crud.* (3) cured. Dr.

Fellows, in "Neurological Clinic" reports following: Boy of thirteen years had epilepsy, treated unsuccessfully with several remedies. Upon the following italicized indications, viz: *Vertigo, followed by falling down in an unconscious state with convulsive movement,* he received *Œnanthe crocato* (3), which apparently cured him in four months. In another case the same symptoms appeared, and the same remedy was given with, apparently, curative results. This last case was marred by the alternation for a short time of *Digitalis* with the *Œnanthe.*

